# Experience of Stigma for Individuals Living with Personality Disorders: A Systematic Review and Meta- Aggregation

**DOI:** 10.1192/j.eurpsy.2026.10223

**Published:** 2026-07-31

**Authors:** A. L. Colonello, C. A. Marshall, S. Sibbald, K. Anderson, H. Stuart

**Affiliations:** 1 Health & Rehabilitation Sciences; 2 School of Occupational Therapy; 3 School of Health Studies & Schulich School of Medicine and Dentistry; 4 Department of Epidemiology & Biostatistics Department of Psychiatry Schulich School of Medicine & Dentistry, Western University, London; 5 Department of Psychiatry and the School of Rehabilitation Therapy, Queens University, Kingston, Canada

## Abstract

**Introduction:**

The stigma literature shows that negative attitudes and discriminatory interactions lead to poorer health experiences and reduced life expectancy.Stigma is ubiquitous and healthcare providers are not immune from the outward internal and systematic manifestations of stigma in their practices. The way health care providers view patients can determine the quality of care that is provided and impact the overall treatment outcomes. There is a dearth of research which synthesizes the experiences of stigma of persons living with personality disorders (PD). No known systematic reviews have focused on the experiences of stigma for persons living with a PD.

**Objectives:**

To synthesize existing qualitative research exploring experiences of stigma for people with PDs and to identify gaps and areas of saturation in the existing current literature.

**Methods:**

We deployed a systematic search of seven academic databases: Medline, PsychINFO, EMBASE, SCOPUS, Nursing and Allied Health Abstracts, Social Work Abstracts, and Sociological Abstracts. We followed the methodology described by the Joanna Briggs Institute (JBI) to complete a systematic review of experience and meta-aggregation. Consistent with the Preferred Reporting Items for Systematic Reviews and Meta-Analyses guidelines, we conducted a title/ abstract screening of every article identified in the search, a full text review, and data extraction followed by a meta-aggregation of the findings of the included studies.

**Results:**

After de-duplication, 6,079 records remained. In total, 48 studies were included in the final review (Figure 1). Half of the included studies (n=24; 50%) represented participants in Europe. Across 47 of these studies, 1,157 participants living with a PD were included. Samples focused primarily on Cluster B PDs, mainly borderline personality disorder (BPD) and antisocial personality disorder (n=1,069), with the remaining participants (n=89) described as general PDs. No studies capturing the distinct experiences of people with other PD diagnoses (e.g., schizoid) were identified. The meta-aggregation yielded 98 extracted themes which were organized into four synthesized findings: 1) the cost of labelling personality: clarity and confusion; 2) (‘ill’) legitimacy, burden, and powerlessness; 3) stigma in health-care settings and its influence on treatment; and 4) resistance to stigma and pathways to recognition: reframing and connecting.

**Image 1:**
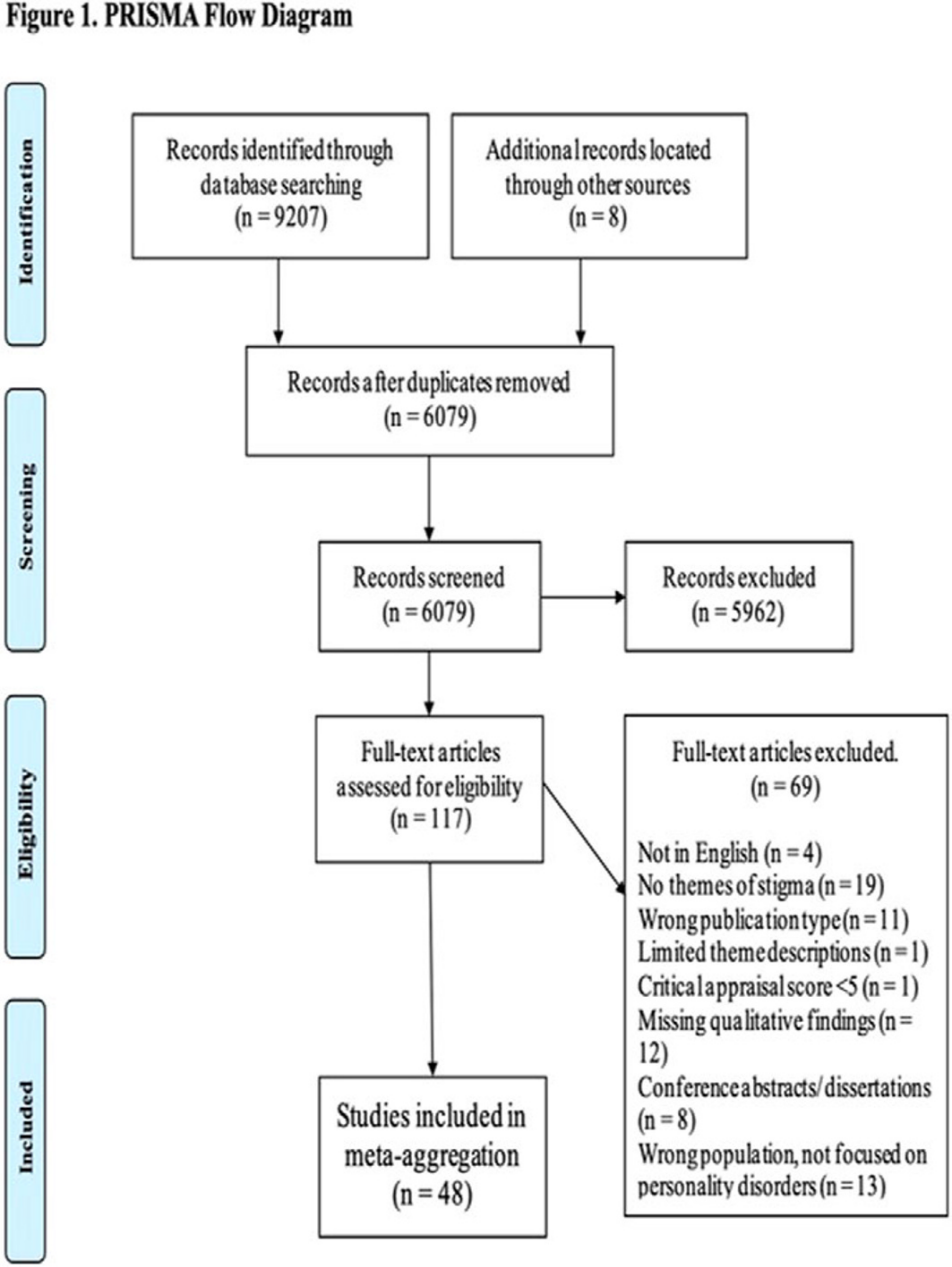
Image 1: Long description.

**Conclusions:**

In this research, we identified a range of gaps and areas of saturation in research exploring the experiences of stigma for individuals living with PDs. Future research should explore the experiences of stigma faced by people with other PDs beyond BPD. We recommend that practitioners increase their awareness of the high degrees of stigma described by individuals living with PDs in the context of healthcare encounters, and revise their practice in ways that mitigate this stigma.

**Disclosure of Interest:**

None Declared

## Long descriptions

### Image 1: Long description

The flowchart is organized into four vertical stages labeled on the left.

1. Identification stage: Two top boxes show Records identified through database searching (n = 9207) and Additional records located through other sources (n = 8). Arrows from both point down to a single box: Records after duplicates removed (n = 6079).

2. Screening stage: An arrow points down to Records screened (n = 6079). A lateral arrow points right to Records excluded (n = 5962).

3. Eligibility stage: An arrow points down to Full-text articles assessed for eligibility (n = 117). A lateral arrow points right to a large box for Full-text articles excluded (n = 69). This box lists specific reasons: Not in English (n = 4), No themes of stigma (n = 19), Wrong publication type (n = 11), Limited theme descriptions (n = 1), Critical appraisal score < 5 (n = 1), Missing qualitative findings (n = 12), Conference abstracts/dissertations (n = 8), and Wrong population, not focused on personality disorders (n = 13).

4. Included stage: A final arrow points down to the bottom box: Studies included in meta-aggregation (n = 48).

Navigate back to Image 1:.

